# Analysis of phosphoinositide 3-kinase inhibitors by bottom-up electron-transfer dissociation hydrogen/deuterium exchange mass spectrometry

**DOI:** 10.1042/BCJ20170127

**Published:** 2017-05-16

**Authors:** Glenn R. Masson, Sarah L. Maslen, Roger L. Williams

**Affiliations:** Medical Research Council, Laboratory of Molecular Biology, Cambridge CB2 0QH, U.K.

**Keywords:** ETD, HDX-MS, HDX-MS/MS, PI3K

## Abstract

Until recently, one of the major limitations of hydrogen/deuterium exchange mass spectrometry (HDX-MS) was the peptide-level resolution afforded by proteolytic digestion. This limitation can be selectively overcome through the use of electron-transfer dissociation to fragment peptides in a manner that allows the retention of the deuterium signal to produce hydrogen/deuterium exchange tandem mass spectrometry (HDX-MS/MS). Here, we describe the application of HDX-MS/MS to structurally screen inhibitors of the oncogene phosphoinositide 3-kinase catalytic p110α subunit. HDX-MS/MS analysis is able to discern a conserved mechanism of inhibition common to a range of inhibitors. Owing to the relatively minor amounts of protein required, this technique may be utilised in pharmaceutical development for screening potential therapeutics.

## Introduction

Hydrogen/deuterium exchange mass spectrometry (HDX-MS) is a technique that allows for the rapid measurement of deuterium incorporation into proteins, providing information on protein structure and solvent accessibility. HDX-MS has recently seen a resurgence in application and has been widely used to determine the basis of protein:protein [1], protein:membrane [2,3] and protein:nucleic acid interactions [4], as well as to determine the nature of conformational changes brought about by other perturbations of the protein, e.g. by mutations [2,5] or by post-translational modification [6]. Additionally, HDX-MS has been greatly used and lauded in the design, manufacture and quality control of biopharmaceuticals [7,8]. The use of HDX-MS to determine the sites of small-molecule binding is a far less routine application compared with the application to biopharmaceuticals, despite possible advantages over competing technologies [9,10]. This may be due to the presence of ‘non-canonical’ effects on ligand binding [11] (the observation that ligand binding may counterintuitively result in an increase in the HDX rate) and the restricted resolution of HDX-MS.

Currently, the majority of HDX-MS is conducted at the ‘peptide’ level of resolution — where observations of solvent exchange are restricted to stretches of amino acids typically between 5 and 20 residues long. The inability of tandem mass spectrometry to, until recently, provide single-amino acid resolution was due to the phenomenon of ‘scrambling’ — where collision-induced dissociation (CID) fragmentation caused a randomisation of the deuterium signal throughout the peptide [12,13], preventing the retention of valuable information. Recently, however, it has been demonstrated that it is possible to produce peptide fragments using electron-transfer dissociation (ETD) or electron-capture dissociation (ECD) in a manner that has very low to negligible levels of scrambling [14–16].

Phosphoinositide 3-kinases (PI3Ks) are one of the most commonly mutated proteins in cancer [17], with the majority of mutations occurring in the catalytic subunit of the Class IA PI3K, p110α. Mutation of p110α results in a hyperactivation of its lipid kinase activity [2], with the overproduction of phosphatidylinositol (3,4,5)-trisphosphate, resulting in aberrant activation of AKT, a master protein kinase that is responsible for cell survival, cell growth and proliferation. As a driver of oncogenesis, p110α, in a complex with its requisite regulatory subunit p85α, has long been a pharmaceutical target [18], with numerous compounds available that inhibit the enzyme by acting as ATP-mimetics, binding to the cleft found at the interface of the N- and C-lobes of the kinase domain. Primarily, X-ray crystallography has been used to determine the mechanisms of compound binding with Class IA PI3Ks [19–22], but in order to facilitate crystallisation, truncated or mutated proteins have been utilised, or the mechanism of compound binding to p110α has been inferred from the crystal structures of different isoforms.

In the present study, we demonstrate how ETD fragmentation can be used to produce single-amino acid resolution HDX-MS on a challenging crystallisation target, and how these data can be used to determine the precise amino acids that are involved in inhibitor binding, providing evidence for how certain compounds confer isoform specificity.

## Experimental methods

### PI3Kα purification

PI3Kα was purified as described previously [2]. Briefly, PI3Kα was expressed in 3 l of *Spodoptera frugiperda* (Sf9) cells at a density of 1.0 × 10^6^ cells/ml by co-infecting with viruses encoding both the catalytic and regulatory subunits. The catalytic subunit was expressed with an N-terminal 6xHis Tag, followed by a tobacco etch virus protease site. After 48 h of infection at 27°C, the cells were sedimented and washed with ice-cold phosphate-buffered saline, prior to being flash-frozen in liquid nitrogen. The cells were then lysed in lysis buffer [20 mM Tris (pH 8.0), 100 mM NaCl, 5% glycerol (vol/vol), 10 mM imidazole and 2 mM β-mercaptoethanol, and one EDTA-free protease inhibitor tablet (Roche) added per 50 ml of buffer] by 3 min of probe sonication. Lysed cells were then centrifuged for 45 min at 140 000 ***g*** at 4°C. The supernatant was then filtered using a 0.45 µm Minisart filter unit (Sartorius Biotech), followed by being passed over a 5 ml HisTrap FF column (GE Healthcare) equilibrated in His buffer A [20 mM Tris (pH 8.0), 100 mM NaCl and 2 mM β-mercaptoethanol]. The column was then washed with 30 mM imidazole in His buffer A, followed by a gradient of 0–100% of His buffer B [20 mM Tris (pH 8.0), 100 mM NaCl, 300 mM imidazole and 2 mM β-mercaptoethanol]. PI3Kα-containing fractions were pooled & passed over a 5 ml heparin HP column (GE Healthcare) which had been equilibrated in Hep buffer A [20 mM Tris (pH 8.0), 100 mM NaCl, and 2 mM DTT). PI3Kα was eluted using a 0–100% gradient of Hep buffer B [20 mM Tris (pH 8.0), 1 M NaCl and 2 mM DTT]. PI3K-containing fractions were then concentrated to a volume of 1.5 ml using an Amicon 50k-centrifugal concentrator (Millipore) and then injected onto a Superdex 16/60 S200 gel filtration column (GE Healthcare) equilibrated using Gel Filtration Buffer [20 mM HEPES (pH 7.4), 100 mM NaCl and 2 mM TCEP]. PI3Kα-containing fractions were then concentrated to 5 mg/ml, aliquoted, flash-frozen in liquid nitrogen and stored at −80°C prior to use.

### Deuterium incorporation

Deuterium incorporation experiments were conducted by incubating 10 µl of 5 µM PI3Kα for 5 min with 40 µl of D_2_O buffer [containing 20 mM HEPES (pH 7.5), 100 mM NaCl, 2 mM TCEP and 1% DMSO in 94.6% D_2_O, for a total final concentration of 75.7% D_2_O]. D_2_O buffer was supplemented with one of four small molecule PI3K inhibitors (as detailed in the Results section): 10 µM GDC-0941, 10 µM GSK2126458, 10 µM ZSTK474 or 200 µM idelalisib (also known as Zydelig, CAL-101 and GS-1101), ensuring a >95% occupancy of the PI3Kα. The exchange reaction was quenched by the addition of 20 µl of Quench Solution (2 M guanidinium chloride and 2.4% formic acid), rapid mixing and immediate flash-freezing in liquid nitrogen. For the 0.005 min time point, the samples were prepared on ice in a 4°C room with pre-chilled D_2_O buffer and pipette tips, and incubated with D_2_O buffer for 3 s. Samples were stored at −80°C prior to analysis. All deuterium-labelled samples were repeated four times.

### Data acquisition

Data were collected using the HDX Manager (Waters, U.K.) set at 0.1°C in-line with a SYNAPT G2-Si HDMS (Waters, U.K.) fitted with an ETD ion source block. In-line peptic digestion was conducted using an Enzymate BEH Pepsin 2.1 × 30 mm Column (Waters, U.K.) at 15°C, where protein was digested for 2 min. Peptides were eluted onto an Acquity 1.7 µm particle, 100 mm × 1 mm C18 UPLC column (Waters, U.K.) in buffer A (0.1% formic acid) with a 12 min 5–36% gradient of buffer B (100% acetonitrile and 0.1% formic acid). Data were acquired over a mass range of 300–2000 *m/z* with a spray voltage of 2.6 kV.

### Peptide identification

Peptide identification was conducted using the ProteinLynx Global Server (PLGS Waters, U.K.). Three samples of non-deuterated PI3Kα were injected, with data collection conducted in MSe mode for the identification of peptide sequences [23]. Peptides with an intensity of over 10 000, a mass error <5 ppm and present in at least two of the three data acquisitions were pooled and imported into the DynamX data analysis software (Waters, U.K.). Any peptides that failed to meet these criteria were excluded from further data analysis. For PI3Kα, a total of 483 peptides were of sufficient quality for analysis, with 99.3% coverage and a mean redundancy of 6.06 per amino acid.

### Mass analysis of peptide centroids

After a first round of automated spectral processing using DynamX, each peptide was individually inspected manually for suitability for further analysis (meeting minimum criteria of spectral envelope clarity and intensity above background noise). No correction was applied for back-exchange as no fully deuterated samples could be obtained. Further data analysis was conducted using the MEMHDX software to test for statistical significance of observed uptake changes that occurred on compound binding [24].

### P1 standard peptide for the determination of low-scrambling ETD conditions

The conditions used for ETD were determined using the standard ‘P1’ peptide (sequence HHHHHHIIKIIK, synthesised by Cambridge Bioscience) as described previously [25]. Briefly, lyophilised P1 peptide was dissolved in 100% D_2_O to a concentration of 100 µM and incubated at room temperature for 24 h in order to fully deuterate the peptide. The peptide exchange reaction was then quenched through dilution by adding 10 µl of peptide to 90 µl of 3% acetonitrile, 0.1% formic acid solution (in H_2_O) and then snap-frozen on dry ice. Immediately prior to analysis, the sample was thawed and injected into the ESI source using a chilled syringe. The ETD chemical reagent used was 1,3-dicyanobenzene (Sigma). Appropriate ETD reagent levels were checked by switching the instrument to the negative ion mode and monitoring the molecular ion at *m/z* 127 until an intensity of >1E × 10^6^ was achieved. Collected *c* and *z* fragment ions were then compared with a theoretical 0% scrambling profile (determined from refs [25–27]) to look for evidence of scrambling. Instrument parameters were adjusted to ensure minimal scrambling. The optimised electrospray source settings were: capillary voltage 2.5, sampling cone 20 kV, source offset 10, source 80°C, desolvation 150°C, cone gas 50 l/h, desolvation gas 300 l/h, make up 25.0 ml/min, Nebuliser 6.0 bar. Instrument settings were: velocity 300 m/s, wave height 0.31 V, trap high gas 22.6, low gas 12.0 and transfer 0.5. Triwave settings were: entrance −3.0, bias 2.0, trap DC −3.0, trap gate −2.0 and exit −5.5. RF settings were: StepWave 300 V, ion guide 300 V, trap ETD 450 and transfer 350. When instrument settings were established that gave no scrambling, the settings were stored and used for the subsequent fragmentation of PI3K peptides.

### Focused ETD

Peptides that showed protection upon compound binding from HDX-MS experiments were subjected to ETD applying a targeted approach. Using an exact mass retention time window, peptides with a charge state of +3 and above were selected for fragmentation by ETD. ETD fragment data were analysed initially using DynamX (Waters), followed by visualisation using Prism 6 (Graphpad) and Pymol.

### Analysis of deuterium scrambling

To determine the levels of scrambling for the individual peptides analysed, we initially used the neutral loss of ammonia upon fragmentation as a gauge, as described previously [28]. Briefly, in the case of 0% scrambling, the mass of deuterium associated with the intact peptide (*D*_pept_) would be equal to that of the same peptide when the ammonia is removed (*D*_pept-NH3_). We conducted a Student's *t*-test to determine whether there was a significant difference between *D*_pept_ and *D*_pept-NH3_. Additionally, we also determined the level of scrambling throughout the peptide by comparing the theoretical 100% random scrambling mass of each fragment with the fragments' observed masses. First, the number of possible ‘scrambling sites’ (Total_ss_) for the entire peptide is determined as follows:

$$\mathrm{Tota}l_{\mathrm{ss}}=3+z+n_{\mathrm{NH}}+n_{\mathrm{labile}\;\mathrm{hydrogens}}$$

where *z* is the charge state of the intact peptide, *n*_NH_ is the number of amide groups and *n*_labile hydrogens_ are the hetero-atom bound hydrogens found on the side chain groups that are prone to scrambling (Ser/Thr/Cys/Tyr/Typ/Asp/Glu/His have one labile hydrogen, Asn/Lys/Gln have two and Arg has four; the three at the start of the equation refers to the NH_3_^+^ group found at the N-terminus of the peptide). To determine the mean amount of incorporated mass that could be expected given 100% scrambling, the mean expected mass for each site was defined as *D*_pept_/Total_ss_. The number of scrambling sites for any given *c*_n_ ion (*c* ion_ss_) can be calculated by using (eqn 1), but for a *z_n_* ion, however, the formula is as follows:

$$z\;\mathrm{io}n_{\mathrm{ss}}=1+z+n_{\mathrm{NH}}+n_{\mathrm{labile}\;\;\mathrm{hydrogens}}.$$

The theoretical 100% scrambled mass for any given fragment is then:

$$c\;\;\mathrm{or}\;\;z\;\mathrm{io}n_{\mathrm{ss}}\;*\;\left(\frac{D_{\mathrm{pept}}}{\mathrm{Tota}l_{\mathrm{ss}}}\right)=100\text{\%}\;\;\mathrm{scrambled}\;\;\mathrm{mass}.$$

To determine whether the observed fragment masses are significantly different from the theoretical scrambled masses, a pairwise ANOVA is conducted.

### Note on variation, error handling and propagation

All ETD experiments were conducted as four biological repeats — each exchange reaction was conducted independently and their level of deuterium incorporation was measured separately. All errors stem from the biological variation associated with these repeats. Typically, the standard deviations for each fragment are <0.05 Da. To determine single-amino acid resolution, the mass of peptide fragment ‘*n*’ was subtracted from the ‘*n* + 1’ fragment, with the resulting single-amino acid mass having an estimated variance of the sum of the variances of the ‘*n*’ and ‘*n* + 1’ fragments. When comparing two states, for example unbound and bound, the two single-amino acid masses are compared, resulting in the sum of their variances (assuming that the two quantities are not correlated). This propagation of error during analysis may exaggerate the uncertainty associated with the differences between two states.

## Results

### Identification of the compound-binding sites

To determine where compounds bound to PI3Kα, we conducted HDX-MS experiments using recombinantly expressed PI3Kα purified from insect Sf9 cells (as described previously [2]). Using this material, we screened four compounds: ZSTK474, a pan-specific Class I PI3K ATP-competitive inhibitor with a reported IC_50_ for PI3Kα of 16 nM [29]; GSK2126458 (also known as Omipalisib and GSK458), an inhibitor with a reported *K_i_* of 0.019 nM [30]; GDC-0941, a Class I selective inhibitor with a reported IC_50_ of 3 nM [31] and CAL-101 (also known as idelalisib, GS-1101 and Zydelig), a p110δ-specific inhibitor, which has a reported IC_50_ of 8600 nM for PI3Kα (IC_50_ for PI3Kδ 19 nM) [20].

**Figure 1.**
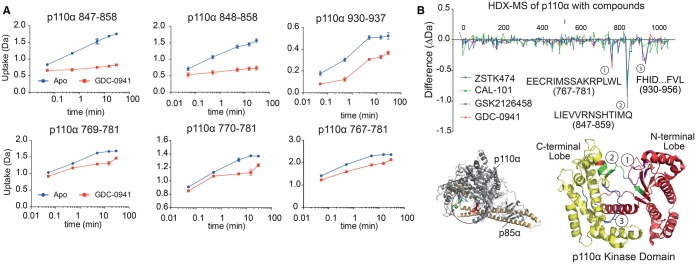
Standard-resolution HDX-MS detailing compound binding. (**A**) HDX as a function of deuteration time for peptides showing a reduction in solvent exchange occurring in p110α upon incubation with 10 µM GDC-0941. The optimum time point for HDX-MS/MS acquisition was determined to be 5 min. Averages shown from three replicates, with error bars representing standard deviations. Some error bars are smaller than the markers. (**B**) Difference plots for all four compounds binding to p110α after 5 min deuteration. Markers are placed at the positions of the midpoints of each peptide. Peptide locations are also marked on the X-ray structure (below) for the p110α (shown in grey)/p85α (shown in orange) heterodimer. Compound binding caused greatest decreases in solvent exchange in three peptides, residues 767–781 (EECRIMSSAKRPLWL, shown in pink, labelled 1), residues 847–859 (LIEVVRNSHTIMQ, shown in green, labelled 2) and residues 930–956 (FHIDFGHFLDHKKKKFGYKRERVPFVL, shown in blue, labelled 3).

A standard-resolution HDX-MS experiment was first conducted to establish an optimum duration of deuterium incorporation in order to determine compound binding, where there is a maximum difference between the bound and unbound exchange. Experiments were conducted where PI3Kα was incubated with deuterium for 3, 30, 300, 900 or 1800 s (Figure 1A) in the presence of 10 µM GDC-0941. From this analysis, we determined that a 5 min deuterium exposure time was optimal to observe changes in solvent exchange associated with compound binding for individual peptides.

To determine which peptides are involved, an initial HDX-MS experiment was conducted with each of the four compounds (GDC-0941, GSK2126458, ZSTK474 or CAL-101). Each compound was screened at saturating concentrations to determine areas of interaction using standard-resolution HDX-MS. Three of the compounds (GDC-0941, GSK2126458 and ZSTK474) have nanomolar affinities and were incubated with PI3K in a 1:2 molar ratio to ensure their occupancy, whereas CAL-101 (also known as idelalisib), a p110δ isoform-specific PI3K inhibitor [20], had to be present at a 40× excess to ensure >95% occupancy. This initial HDX-MS experiment showed that all four compounds bound in approximately the same manner — in the ATP-binding cleft found between the two lobes of the kinase domain (see Figure 1B). All compounds caused a reduction in solvent exchange within three main areas of PI3Kα: residues 767–781 (EECRIMSSAKRPLWL), residues 847–859 (LIEVVRNSHTIMQ), which includes the hinge between the N- and C-lobes of the kinase domain, and residues 930–956 (FHIDFGHFLDHKKKKFGYKRERVPFVL), which includes most of the ‘activation loop’ (Figure 1B).

### Determination of low-scrambling conditions

To determine fragmentation conditions resulting in minimal scrambling, we used a model ‘P1’ peptide as described previously [25] to determine scrambling levels. From this analysis, we determined that scrambling levels were <10%.

Targeted ETD was conducted for 30 s periods for six peptides that decreased in exchange upon compound binding (767–781, 769–781, 770–781, 848–858, 848–849 and 930–937). Partial *c* and *z* ion series could be constructed for all peptides except that of peptide 930–937, which failed to fragment efficiently. Successful ETD fragmentations were most readily achieved with peptides having a charge state of +3 or above and that were sufficiently abundant, typically over 1E × 10^5^.

### Single-amino acid resolution of peptide 848–858

**Figure 2.**
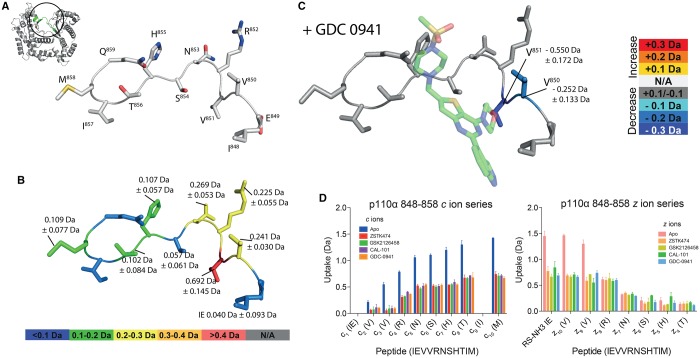
HDX-MS/MS of residues 848–858, detailing compound binding. (**A**) Location of residues 848–859, with side chains shown as sticks. (**B**) HDX-MS/MS data showing the amount of deuterium incorporation after 5 min exposure, coloured according to uptake. (**C**) Single-amino acid resolution hydrogen/deuterium exchange detailing reductions in the solvent exchange rate that occur upon GDC-0941 binding. (**D**) Deuterium uptake of *c* and *z* ions of peptide 848–858 with and without the four compounds. Error bars are the standard deviation across four replicate measurements of each fragment.

Peptide 848–858 (sequence IEVVRNSHTIM; see Figure 2A–D), found in the hinge region of the kinase domain of PI3Kα, provided fragment ions *c*_2_*–c*_8_, *c*_10_ and *z*_4_–*z*_10_ and the charge-reduced NH_3_-deficient species (RS-NH_3_; see Figure 2D). Single-amino acid resolution HDX-MS was afforded by comparing the mass of consecutive fragments in either series, allowing for the estimation of individual amino acid HDX (Figure 2B). The total peptide mass difference of the 848–858 precursor peptide was −0.70 ± 0.05 Da on the addition of 10 µM GDC-0941. Using HDX-MS/MS (hydrogen/deuterium exchange tandem mass spectrometry), this change was localised to two amino acids, Val^851^ (−0.55 ± 0.17 Da) and Val^850^ (−0.25 ± 0.13 Da) (*z* ion series; Figure 2C), through observations of the mass differences between the bound and unbound exchange rates of the *z*_9_ and *c*_3_ ions of the fragment ion series (see Figure 2D). All four compounds exhibited similar levels of protection for Val^851^, a reduction in ∼0.5–0.7 Da. However, the reduction on Val^850^ was not seen with either CAL-101 or ZSTK474. CAL-101 uniquely caused a protection on Asn^853^ of 0.11 ± 0.13 Da. The method used to calculate the error associated with these measurements can be found above and may artificially inflate the perceived inaccuracy of the data.

### Single-amino acid resolution of peptide 769/770–781

**Figure 3.**
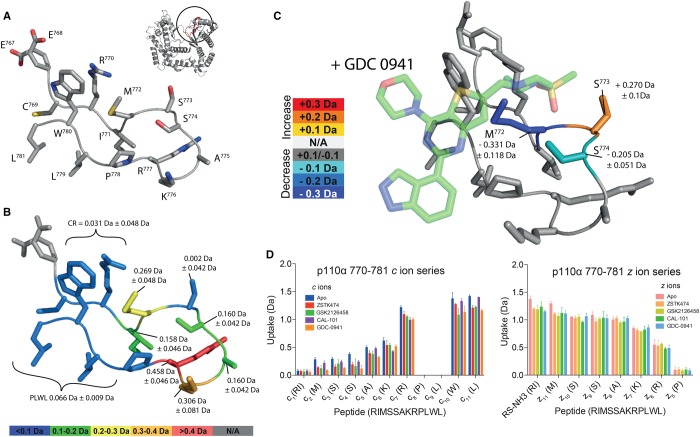
HDX-MS/MS of residues 770–781, detailing compound binding. (**A**) Location of residues 767–781, with side chains shown as sticks. (**B**) HDX-MS/MS data showing the amount of deuterium incorporation after 5 min exposure, coloured according to uptake. (**C**) Single-amino acid resolution hydrogen/deuterium exchange detailing reductions in the solvent exchange rate that occur upon GDC-0941 binding. (**D**) Deuterium uptake of *c* and *z* ions of peptide 767–781 with and without the four compounds. Error bars are the standard deviation across four replicate measurements of each fragment.

Peptide 770–781 (sequence RIMSSAKRPLWL; see Figure 3A–D), found in the hinge region of the kinase domain of PI3Kα, provided fragment ions *c*_1_*–c*_7_, *c*_10_*–c*_11_ and *z*_5_–*z*_11_ and RS-NH_3_ (see Figure 3D). Peptide 769–781 (sequence CRIMSSAKRPLWL) produced a *z*_5_–*z*_12_ series and an RS-NH_3_. Single-amino acid resolution HDX-MS was afforded by comparing the mass of consecutive fragments in either series, allowing for the determination of individual amino acid HDX (Figure 3B). The total peptide mass difference of the 769–781 precursor peptide was −0.34 ± 0.04 Da on the addition of 10 µM GDC-0941, and for 770–781, it was −0.24 ± 0.05. Using HDX-MS/MS on peptide 769–781, this net change of 0.24 Da upon GDC-0941 binding was determined to be a composite of both reductions and increases in solvent exchange rates, localised on three amino acids within the peptide: Met^772^ (−0.33 ± 0.12 Da), Ser^773^ (+0.27 ± 0.10 Da) and Ser^774^ (−0.21 ± 0.05 Da) (*z* ion series). The changes in exchange rate were conserved in peptide 770–781, although with a smaller magnitude: Met^772^ (−0.18 ± 0.06 Da), Ser^773^ (+0.03 ± 0.05 Da) and Ser^774^ (−0.76 ± 0.06 Da) (*c* ion series).

### Determination of scrambling levels

**Figure 4.**
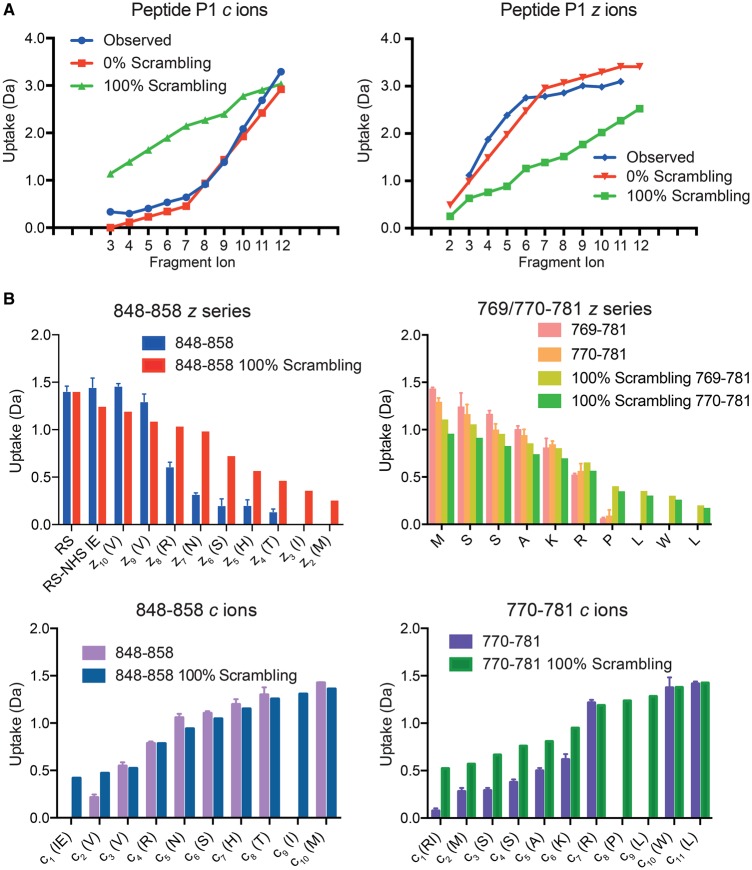
Determination of scrambling. (**A**) The test peptide, peptide P1, *c* and *z* ions. The theoretical values for 100% (green) scrambling and 0% (red) non-scrambled are shown with the measured values (blue). (**B**) The *z* and *c* ions for a variety of peptides with their theoretical 100% scrambled values. Error bars are the standard deviation across four replicate measurements of each fragment.

To determine the degree of scrambling in the data obtained from the fragment ions, two methods were used. Initially, we determined whether there was a significant difference between the deuterium content of the intact precursor peptide (*D*_pept_) and the NH_3_-deficient adduct (*D*_pept-NH3_) as proposed previously [28]. However, due to levels of deuterium incorporated into the peptides under investigation, the differences in masses between *D*_pept_ and *D*_pept-NH3_ even under 100% scrambling would be within experimental error. For example, peptide 770–781 has a mean deuterated uptake of 1.4 ± 0.04 Da at 5 min incubation. If this peptide was to be subjected to 100% scrambling conditions, the associated ammonia loss ion could be expected to have a deuterated mass of 1.28 Da (under our low-scrambling conditions, the actual ammonia loss ion observed a mass of 1.41 ± 0.04 Da, suggesting that no scrambling had occurred). It is conceivable that with many peptides, the mass attributable to this ammonium group would be <0.1 Da — similar to the variability between repeats (see Figure 4A,B). To circumvent this, we determined the theoretical 100% scrambling mass of any given fragment given the intact mass deuterium level, and compared the predicted pattern of 100% scrambled fragment masses with those observed, and then determined whether there was a significant difference between the two. Alternatively, the use of an equilibrium labeled control sample, as previously suggested [25], could alleviate this problem. Additionally, in scrambling conditions, any reduction in solvent exchange would be distributed evenly throughout the entire peptide — rather than focused on individual amino acids. Given that the reductions in solvent exchange are seen reproducibly on single residues, this provides further evidence that these experiments were conducted under low-scrambling conditions.

## Discussion

During the process of lead generation in the development of new pharmaceuticals, it is often vital to determine the region in which the compound is interacting with the target. Typically, two techniques are used for this purpose, X-ray crystallography and NMR. Both of these techniques require significant amounts of protein and have limitations on their applicability, such as the difficulties associated with promoting crystallography with intrinsically disordered proteins, or deciphering the spectra of proteins greater than 50 kDa in NMR. Here, we present a technique that can provide insights into the mechanism of compound binding at a single-amino acid resolution using HDX-MS, coupled with ETD fragmentation.

HDX-MS has *prima facie* many possible advantages for compound screening: there is no size limitation on the protein being screened and there are no labelling requirements (other than the isotope substitution of deuterium for hydrogen); compounds with modest affinities can be used in moderate concentrations to ensure occupancy and the measurement is conducted in solution. However, traditional HDX-MS approaches are limited in their resolution by the phenomenon of ‘scrambling’ — the propensity for the hydrogen atoms associated with the precursor peptide to randomise during CID fragmentation methods, preventing the determination of their positions through MS/MS [13]. Recently, however, it was found that scrambling could be effectively reduced to 0% through the use of ETD and ECD fragmentation techniques [27,32].

Using the ‘P1’ peptide (sequence HHHHHHIIKIIK) as a measure of scrambling propensity (as previously described [25]), we could determine optimum conditions for our instrument to minimise scrambling (see Figure 4A). We determined the predicted masses of every fragment under scrambling conditions and compared them with our observed values (see Figure 4B). From this comparison, along with the differences in masses between the ammonia losses and intact peptide masses, we could be confident in our low-scrambling conditions. Additionally, the trends in deuteration in the *c* ion series of the overlapping 769–781 and 770–781 peptides closely agree.

In our study, using full-length constructs for both the regulatory and catalytic subunits of the p110α isoform, HDX-MS identified the binding sites for all four compounds in solution, including that of the low-affinity CAL-101. The region identified is consistent with previous crystallographic observations of various PI3K isotypes, using the three tight binding compounds: GDC-0941 binding to p110β [33], p110δ [22] and p110γ [31]; ZSTK474 binding to p110δ [22]; GSK2126458 binding to p110γ [30] and CAL-101 binding to p110δ [20].

ETD fragmentation technology allowed us to conduct HDX-MS/MS, producing single-amino acid resolution HDX data for three peptides that covered this area of interaction in the kinase domain cleft of PI3Kα. Generally, successfully fragmenting peptides had a charge of 3 or greater and were of a reasonable abundance (greater than 1E × 10^5^). The observed reduction in exchange for Val^851^ was conserved for all compounds, with the magnitude for the reduction in exchange varying from 0.55 Da for GDC0941 to 0.71 Da for CAL-101. Additionally, Val^850^ has reductions in exchange upon the addition of only two of the compounds; GSK2126458 has a reduction in −0.22 ± 0.16 Da and GDC-0941 causes a −0.25 ± 0.13 Da reduction. The exchange rate of Val^850^ was unaltered upon the addition of either CAL-101 or ZSTK474.

The crystal structure of p110δ bound to CAL-101 indicated the presence of two hydrogen bonds between the purine group N3 and N9 nitrogens of CAL-101 and the backbone amide of Val^828^ (corresponding to Val^851^ of p110α) and backbone carbonyl group of Glu^826^ (corresponding to Glu^849^ of p110α) [20]. HDX-MS (and HDX-MS/MS) measures solvent exchange of backbone amide hydrogens, so it is not surprising that there is a large reduction in solvent exchange observed upon formation of a backbone amide hydrogen bond between N3 of CAL-101 and Val^851^. There is no reduction in solvent exchange of Glu^849^ associated with CAL-101 binding; indeed, there is an increase in +0.23 ± 0.14 Da in solvent exchange. This may be evidence for a less common ‘non-canonical’ effect of compound binding on HDX-MS. It has been proposed that compound binding might, in some cases, increase the occupancy of locally unfolded states [11]. It has been proposed that CAL-101 may have a preference for binding to the p110δ isotype due to this isotype being more readily able to change conformation and accommodate the compound [22]. This non-canonical effect on p110α may be a consequence of the compound forcing a conformational change on the enzyme.

In the 769–781 region, two peptides fragmented adequately to provide single residue HDX, allowing for the observation that three residues, Met^772^, Ser^773^ and Ser^774^, have the largest changes in exchange rate on compound binding. The same pattern of Met^772^ reduction in exchange, Ser^773^ increase, followed by a Ser^774^ decrease was seen for all compounds (GDC-0941, GSK2126458 and ZSTK474), although the magnitude of these changes was much diminished for CAL-101, suggesting a difference in binding mechanism. Using standard resolution, HDX-MS would produce an observation of a 0.240 Da reduction upon GDC-0941 binding, but HDX-MS/MS showed this to be a result of both reductions and increases in the solvent exchange rate on individual residues. The alterations in exchange may be due to Met^772^ moving in and out of the of the ‘specificity pocket’ — a pocket formed between Trp^780^ and Met^772^ that is thought to allow certain ‘propeller’ compounds — such as CAL-101, to be selective for p110δ where the pocket is exaggerated. The fact that there is little difference in exchange in this region on CAL-101 binding may suggest that a more rigid pocket in p110α does not readily undergo a conformational change to accommodate CAL-101's ‘propeller’ into the specificity pocket [20]. Instead, the compound may be accommodated in p110α by other conformational changes that are less energetically favourable than the conformational changes that p110δ can undergo in the specificity pocket.

The utility in HDX-MS/MS may be for identifying potential backbone amide-binding sites that could be exploited by pharmaceuticals. The largest reductions in solvent exchange were observed on Val^851^, which can already be determined to be ‘receptive’ to the formation of a hydrogen bond as it has a global exchange rate of 0.69 ± 0.15 Da. Smaller alterations in the solvent exchange rate that accompany compound binding may be indicative of backbone rearrangements rather than the formation or breaking of hydrogen bonds. The ability of HDX-MS/MS to screen low-affinity compounds [34], as described here with CAL-101, makes the technique an attractive tool for the lead generation process.

A disadvantage of HDX-MS/MS is the inability to identify interactions that are mediated entirely through side chains that have no discernable effect on backbone organisation (this can be overcome, however, through the use of gas-phase HDX-MS, [35]). In addition to this, while it is possible to achieve sufficient very low scrambling with ETD fragmentation to allow for single-amino acid resolution with certain peptides, not all peptides are amenable to fragmentation. None of the peptides that resulted from pepsin digestion of the DFG loop of PI3Kα (residues 930–956) could be adequately fragmented — most probably due to their low abundance. This may be remedied through the use of alternative proteases to produce an alternative selection of peptides that may be of a higher abundance.

In the present study, we have illustrated that by using ETD fragmentation to enable HDX-MS/MS, it is possible to produce single-amino acid resolution solvent exchange data with minimal scrambling. Given the low protein requirements, the sensitivity of HDX-MS to low-affinity compounds and the extra insight provided by amino acid resolution, HDX-MS/MS could be amenable to the medium-throughput screening of compound series within a pharmaceutical setting.

## Abbreviations

AKT: protein kinase B
ANOVA: analysis of variance
CID: collision-induced dissociation
DTT: dithiothreitol
ECD: electron-capture dissociation
ESI: electro-spray ionization
ETD: electron-transfer dissociation
HDX-MS: hydrogen/deuterium exchange mass spectrometry
HDX-MS/MS: hydrogen/deuterium exchange tandem mass spectrometry
PI3K: phosphoinositide 3-kinase
Sf9: *Spodoptera frugiperda*
TCEP: tris(2-carboxyethyl)phosphine
